# Prediction of deep vein thrombosis by ultrasonography and D-dimer in Asian patients with ischemic stroke

**DOI:** 10.1186/s12883-020-01842-w

**Published:** 2020-06-27

**Authors:** Sang Hee Ha, Yeon-Jung Kim, Sung Hyuk Heo, Dae-il Chang, Bum Joon Kim

**Affiliations:** 1 Department of Neurology, Kyung Hee University Hospital, Seoul, Republic of Korea; 2 Department of Neurology, Asan Medical Center, University of Ulsan College of Medicine, 88 Olympic-ro 43-gil, Songpagu, Seoul, 138-736 Republic of Korea

**Keywords:** D-dimer, Deep vein thrombosis, Ultrasonography, Ischemic stroke

## Abstract

**Background:**

Deep vein thrombosis (DVT) is an important complication of ischemic stroke, although the incidence of DVT is regarded as being lower in Asian than in non-Asian patients. Here, we investigated the incidence and factors associated with DVT in Asian patients with ischemic stroke.

**Methods:**

Acute ischemic stroke patients received lower extremity ultrasonography (LEUS) to diagnose the presence of DVT. Clinical characteristics and laboratory results, including D-dimer level, were compared between patients with and without DVT. Independent risk factors for DVT were investigated using multivariable analysis. Similar analysis was performed to identify factors associated with elevated D-dimer level (> 0.5 mg/dl) in acute ischemic stroke patients.

**Results:**

During the study period, 289 patients were enrolled, and 38 (13.1%) showed DVT. Female sex (OR = 2.579, 95% CI = 1.224–5.432; *p* = 0.013) and a high National Institutes of Health Stroke Scale (NIHSS) score (OR = 1.191 95% CI = 1.095–1.294; *p* = 0.005) were independently associated with the presence of DVT, although D-dimer level was not. Stroke mechanism, especially cardioembolic stroke (OR = 3.777, 95% CI = 1.532–9.313; *p* = 0.004; reference: large artery atherosclerosis), NIHSS score (OR = 1.087, 95% CI = 1.002–1.179; *p* = 0.001) and thrombolysis (OR = 12.360, 95% CI 2.456–62.213; *p* = 0.002) were independently associated with elevated abnormal D-dimer levels.

**Conclusion:**

The severity of ischemic stroke, but not the D-dimer level, was associated with the presence of DVT in Asian ischemic stroke patients. D-dimer level was influenced by the stroke mechanism. LEUS in patients with severe neurological deficit, rather than screening with D-dimer, may be more beneficial for diagnosing DVT in Asian patients with acute ischemic stroke.

## Background

Ischemic stroke survivors demonstrate a high rate of neurological deficit, with a considerable proportion of patients remaining immobilized for a period after stroke. Immobility after ischemic stroke can cause various complications, such as deep vein thrombosis (DVT), pressure ulcers, or sarcopenia leading to falls and fractures [1]. DVT and pulmonary embolism are leading causes of death after stroke [2] and are the most common causes of death during the period of active rehabilitation, [3] with newly-developed DVT increasing the rate of mortality at 3 months after stroke [4].

The incidence of DVT differs according to the modality used to detect its presence after stroke, [5] with previous studies reporting prevalence rates ranging from 20 to 70%. Several studies have shown that the incidence of DVT may differ according to ethnicity and race, with the incidence of DVT being lower than expected in studies on Asian patients with stroke (4.8 to 45%) [6]. The prevalence of DVT in the general population is also lower in Asian populations than in non-Asian patients [7]. However, the annual incidence of DVT in the general population is increasing in Asia, [8] and DVT remains an important issue for determining the treatment strategy for stroke, especially regarding the use of antithrombotics.

Although the D-dimer level may be useful for detecting DVT in various conditions, the initial D-dimer level has shown limitations for predicting the presence of DVT among acute ischemic stroke patients [9]. In the present study, we used lower extremity ultrasonography (LEUS) to evaluate the prevalence of DVT after ischemic stroke in Korean patients and investigated the factors associated with the presence of DVT to reveal the optimal strategy for identifying DVT in Asian stroke patients.

## Methods

### Patients and study design

Acute ischemic stroke patients within 7 days from stroke onset who were admitted to our stroke center between January 2014 and December 2016 were screened. Among them, those who were confirmed to have ischemic stroke according to diffusion-weighted imaging and who also received LEUS were consecutively enrolled. LEUS was performed to screen for concomitant peripheral artery disease or DVT. The data on peripheral artery disease were published previously [10]. Patients with poor image quality, without LEUS data, those under hormone replacement therapy, those who were pregnant, and those who were immobilized previous to the index stroke were excluded from the analysis.

The clinical data were obtained from a prospectively registered stroke registry. Active cancer was defined as histologically confirmed cancer receiving active treatment or diagnosed within the past 6 months, or recurrent/metastatic cancer. The neurological severity was evaluated by the National Institutes of Health Stroke Scale (NIHSS) scores examined by experienced vascular neurologists, initially at the time of admission. The component of arm and leg weakness of NIHSS score was also presented, separately. The functional status prior to the index stroke was investigated by modified Rankin Scale (mRS). All laboratory data except for lipid profile and D-dimer level were checked on the day of admission to the emergency room using a latex agglutination assay technique. The lipid profile and D-dimer level were checked on the second day of admission after at least 8 h of food deprivation. A D-dimer level ≥ 0.5 mg/L was regarded as abnormal. The mechanism of the stroke was classified according to the TOAST (Trials of Org 10,172 in Acute STroke) classification at discharge [11]. This study was approved by the local ethics committee, and the need for informed consent was waived owing to its retrospective design.

### Lower extremity ultrasonography

The patients underwent LEUS within 3 days of admission, with the procedure being conducted by an experienced radiologist blinded to the clinical data, according to a previous protocol, and regardless of the D-dimer level. A high-resolution 7.5 MHz linear-array transducer was used. Data were obtained from the bilateral femoral, popliteal, peroneal, and posterior tibial veins using B-mode and color Doppler mode. A compression test was performed from the common femoral vein to the ankle, evaluating the posterior tibial and peroneal veins in the calf, and color and spectral Doppler imaging were performed on the femoral vein. Acute DVT was considered positive if a heterogeneous thrombus was present inside any of the screened veins on B-mode, or when non-compressibility or a color Doppler flow signal defect was observed [12].

### Statistical analysis

First, the prevalence of DVT among the ischemic stroke patients was investigated. Then, the clinical characteristics and laboratory results were compared between those with and without DVT. Multivariable analysis using a binary logistic regression model was used to verify the independent risk factors for DVT in acute ischemic stroke patients. A similar analysis was performed between patients with abnormal and normal D-dimer levels. Pearson’s chi-square tests or Fisher’s exact tests were used for categorical variables as appropriate, and Student’s *t*-test was used for continuous variables. Variables identified as having a potential association (*p* < 0.1) in a univariate analysis were entered into each multivariable analysis model. Statistical significance was defined as *p* < 0.05 (two-tailed). All statistical analyses were performed using SPSS 21.0 (IBM Corporation, Armonk, NY).

## Results

During the study period, 326 patients in the acute phase within 7 days from stroke onset were admitted to the stroke center of Kyung Hee University Hospital. Of these patients, 37 were excluded because of poor neuroimaging quality (*n* = 5) or the absence of LEUS data (*n* = 32). Finally, 289 patients were analyzed. The D-dimer level was checked in 283 patients. The mean age of the patients enrolled in the study was 68.1 ± 11.3 years, and 175 (60.6%) of the patients were male. None of the patients were pregnant or under contraceptive or other hormone replacement treatment. One patient was diagnosed with anti-phospholipid syndrome, but did not show a DVT. Two patients showed mRS 4 prior to the index stroke with moderate disability, which needs assistant to walk, but did not show DVT.

### Factors associated with DVT

**Fig. 1:**
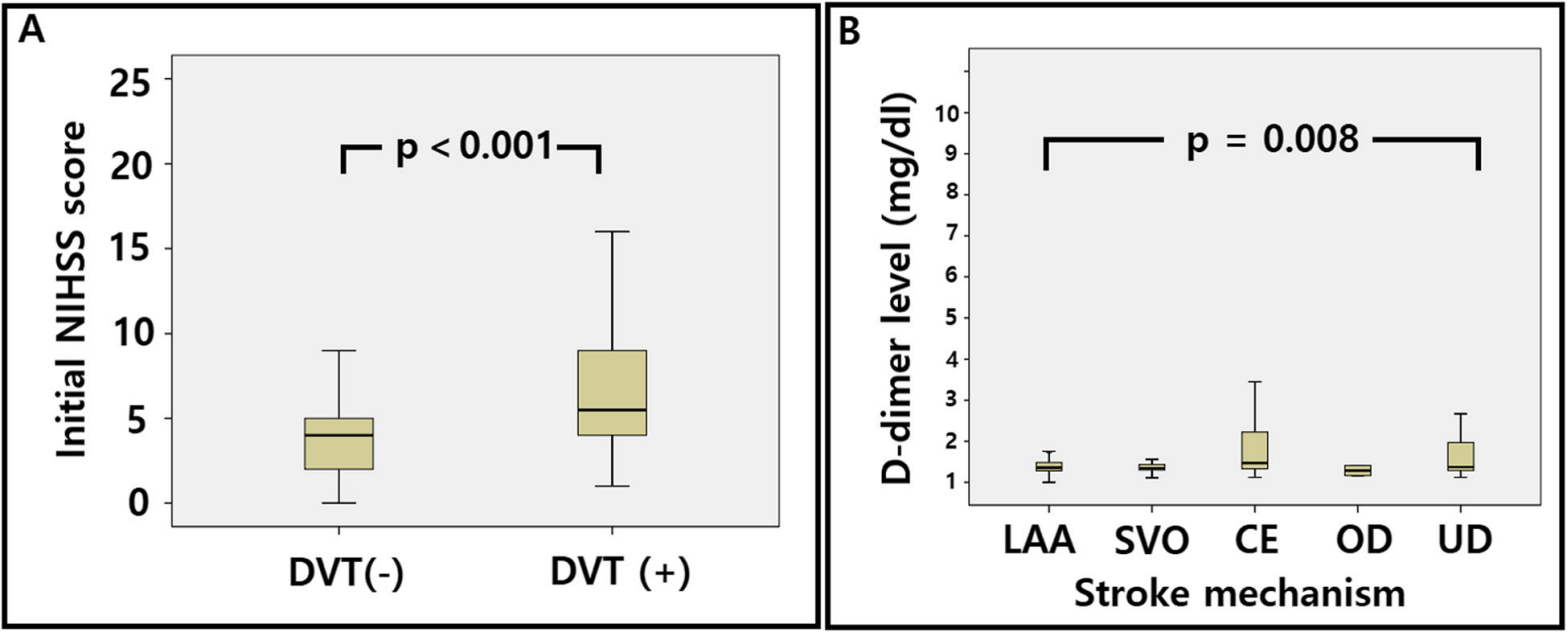
Initial NIHSS according to the presence of DVT (**a**) and D-dimer level according to stroke mechanism (**b**). DVT: deep vein thrombosis, NIHSS: National Institute of Health Stroke Scale, LAA: large artery atherosclerosis, SVO: small vessel occlusion, CE: cardioembolism, OD: other determined, UD: undetermined

Among the enrolled patients, 38 (13.1%) had DVT; 24 with proximal DVT and 14 with distal DVT. The patients with DVT showed a higher proportion of females than those without (60.5% vs. 36.3%, respectively; *p* = 0.004, Table 1). There was no significant difference in risk factors, concomitant disease, or stroke mechanism between those with and without DVT. Patients with DVT showed a higher neurological severity, as represented by a higher NIHSS score on admission (7.4 ± 5.4 vs. 4.1 ± 3.3, respectively; *p* < 0.001; Fig. 1a). Especially, the NIHSS score of lower extremity was different between those with DVT and those without (2.0 ± 1.1 vs. 0.8 ± 1.0, respectively; *p* < 0.001). However, there was no significant difference in the use of antithrombotics and laboratory results between the two groups, including the D-dimer level.

**Table 1:** Characteristics of the patients with and without deep vein thrombosis

	DVT (+) *n* = 38	DVT (−) *n* = 251	*p*-value
Age (years)	71 ± 12	68 ± 11	0.058
Male	15 (39.5%)	160 (63.7%)	0.004
Hypertension	30 (78.9%)	178 (71.2%)	0.321
Diabetes	15 (39.5%)	72 (28.8%)	0.182
Hyperlipidemia	23 (60.5%)	172 (68.8%)	0.310
Smoking	14 (36.8%)	116 (48.4%)	0.270
Previous stroke	6 (15.8%)	39 (15.5%)	0.968
Peripheral artery disease	16 (42.1%)	92 (36.7%)	0.517
Active cancer	4 (1.6%)	1 (2.6%)	0.647
Stroke mechanism			
Large artery atherosclerosis	15 (39.5%)	65 (28.0%)	0.132
Small vessel disease	8 (21.1%)	87 (34.8%)	
Cardioembolism	6 (15.8%)	31 (12.4%)	
Other determined	1 (2.6%)	1 (0.4%)	
Undetermined	8 (21.1%)	66 (26.4%)	
BMI (kg/m^2^)	23.9 ± 3.5	23.9 ± 2.6	0.927
mRS before index stroke	0.1 ± 0.4	0.2 ± 0.7	0.220
Initial NIHSS	7.4 ± 5.4	4.1 ± 3.3	< 0.001
NIHSS arm	1.3 ± 1.2	1.0 ± 1.0	0.160
NIHSS leg	2.0 ± 1.1	0.8 ± 1.0	< 0.001
Laboratory results			
AST (mg/dL)	40 ± 4	41 ± 5	0.732
ALT (mg/dL)	46 ± 15	45 ± 13	0.428
BUN (mg/dL)	18 ± 10	17 ± 5	0.342
Creatinine (mg/dL)	0.8 ± 0.2	0.8 ± 0.4	0.297
D-dimer (mg/dL)	1.2 ± 2.4	0.8 ± 1.2	0.123
FDP (μg/mL)	7.3 ± 13.4	5.8 ± 9.0	0.433
Thrombolysis	2 (5.3%)	12 (4.8%)	0.897
Concomitant antithrombotics			
Single antiplatelet	30 (78.9)	194 (77.3)	0.765
Dual antiplatelet	4 (10.5)	36 (14.3)	
Anticoagulation	4 (10.5)	21 (8.4)	

*DVT* Deep vein thrombosis, *BMI* Body mass index, *mRS* Modified Rankin scale, *NIHSS* National Institutes of Health Stroke Scale, *AST* Aspartate aminotransferase, *ALT* Alanine aminotransferase, *BUN* Blood urea nitrogen, *FDP* Fibrinogen degradation product

According to the results of the multivariable analysis, female sex (odds ratio [OR] = 2.579, 95% confidence interval [CI] = 1.224–5.432; *p* = 0.013) and NIHSS score (OR = 1.191, 95% CI = 1.095–1.294; *p* = 0.005) were independent factors associated with the presence of DVT.

### Factors associated with elevated D-dimer

Of the 283 patients with D-dimer data, 77 (27.2%) showed an abnormal D-dimer level. Patients with an abnormal D-dimer level showed a higher prevalence of active cancer (5.2% vs. 0.5%; *p* = 0.007, Table 2) and elevated fibrinogen degradation product levels (10 ± 15 vs. 3 ± 2; *p* < 0.001) and more received thrombolysis (14.3% vs. 1.5%; *p* < 0.001) than those with normal D-dimer level. Patients with elevated D-dimer also showed a more severe neurological deficit than those without an elevated level (5.8 ± 4.5 vs. 4.0 ± 3.2; *p* < 0.001). The mechanism of stroke differed between those with and without elevated D-dimer (*p* = 0.008), with the proportions of patients with cardioembolism (22.1% vs. 9.8%) or an undetermined mechanism (32.5% vs. 22.9%) being higher in those with an elevated D-dimer level (Fig. 1b).

**Table 2:** Characteristics of the patients according to D-dimer level

	Abnormal D-dimer (*n* = 77)	Normal D-dimer (*n* = 206)	*p*-value
Age (years)	68 ± 12	68 ± 11	0.683
Male	50 (64.9%)	122 (59.2%)	0.381
Hypertension	54 (70.1%)	149 (72.3%)	0.715
Diabetes	25 (32.5%)	57 (27.7%)	0.429
Hyperlipidemia	48 (62.3%)	142 (68.9%)	0.293
Smoking	39 (50.6%)	89 (43.2%)	0.263
Previous stroke	12 (15.6%)	31 (15.0%)	.0.911
Peripheral artery disease	30 (39.0%)	77 (37.4%)	0.807
Active cancer	4 (5.2%)	1 (0.5%)	0.007
Deep vein thrombosis	14 (18.2%)	22 (10.7%)	0.092
Stroke mechanism			
Large artery atherosclerosis	18 (23.4%)	60 (29.3%)	0.008
Small vessel disease	17 (22.1%)	76 (37.1%)	
Cardioembolism	17 (22.1%)	20 (9.8%)	
Other determined	0 (0.0%)	2 (1.0%)	
Undetermined	25 (32.5%)	47 (22.9%)	
BMI (kg/m^2^)	24 ± 4	24 ± 3	0.665
mRS before index stroke	0.3 ± 0.8	0.2 ± 0.6	0.218
Initial NIHSS	5.8 ± 4.5	4.0 ± 3.2	< 0.001
Laboratory results			
AST (mg/dL)	41 ± 4.9	41 ± 5.0	0.941
ALT (mg/dL)	46 ± 13	43 ± 14	0.106
BUN (mg/dL)	18 ± 9	17 ± 5	0.414
Creatinine (mg/dL)	0.9 ± 0.3	0.8 ± 0.4	0.318
D-dimer (mg/dL)	2.2 ± 2.1	0.3 ± 0.1	< 0.001
FDP (μg/mL)	10 ± 15	3 ± 2	< 0.001
Thrombolysis	11 (14.3)	3 (1.5)	< 0.001
Concomitant antithrombotics			
Single antiplatelet	54 (70.1)	166 (80.6)	0.150
Dual antiplatelet	13 (16.9)	25 (12.1)	
Anticoagulation	10 (13.)	15 (7.3)	

*DVT* Deep vein thrombosis, *BMI* Body mass index, *mRS* Modified Rankin scale, *NIHSS* National Institutes of Health Stroke Scale, *AST* Aspartate aminotransferase, *ALT* Alanine aminotransferase, *BUN* Blood urea nitrogen, *FDP* Fibrinogen degradation product

The results of the multivariable analysis showed that cardioembolic stroke (OR = 3.777, 95% CI = 1.532–9.313; *p* = 0.004; reference: large artery atherosclerosis), NIHSS score (OR = 1.087, 95% CI = 1.002–1.179; *p* = 0.001) and thrombolysis (OR = 12.360, 95% CI 2.456–62.213; *p* = 0.002) to be independently associated with elevated abnormal D-dimer levels.

## Discussion

In this study, LEUS revealed a 13% incidence of DVT among patients with ischemic stroke. Female sex and a severe neurological deficit were independently associated with the presence of DVT in the acute phase of ischemic stroke. However, the usefulness of D-dimer level for screening DVT in Asian patients with acute ischemic stroke was limited. Elevated D-dimer level was observed in more than a quarter of patients with ischemic stroke, with the mechanism of stroke being particularly associated with elevated D-dimer level.

Although D-dimer level is useful for detecting DVT in other conditions such as liver disease and malignancy, several studies showed that the initial D-dimer level is not associated with the presence of DVT in stroke patients [9]. Ischemic stroke shows associations with thrombotic/fibrinolytic conditions, especially in the case of cardioembolic and cancer-associated strokes [13]. Therefore, the D-dimer level is typically increased in those with cardioembolic strokes [14] and is useful for detecting cancer-associated strokes [15]. In accord with previous studies, our results also showed that D-dimer level was associated with the stroke mechanism, with cardioembolic stroke being an independent factor associated with elevated D-dimer levels. As D-dimer is elevated in acute ischemic stroke patients and as thrombolysis and the stroke mechanism influences the D-dimer level, it may be less useful for detecting DVT in patients with acute ischemic stroke.

Instead, severe neurological deficits were associated with DVT in acute ischemic stroke patients. There was a significant difference in the NIHSS score between those with and without DVT. Especially the NIHSS score of the lower leg weakness, which directly influences the ambulation in their acute period, showed a significant difference. It is well known that immobilization or paralysis of the lower extremities is associated with DVT because of the increased venous stasis [16]. The prevalence of DVT or pulmonary embolism is lower in Asians than in non-Asian subjects, and coagulopathy is less associated with ischemic stroke in Asian patients [17, 18]. Therefore, the relative importance of systemic coagulopathy represented by the D-dimer level may be less important in Asian patients with ischemic stroke, and immobilization and severe neurological deficit may be more important in the development of DVT in Asian patients with acute ischemic stroke [19].

The results of our study indicate that the role of D-dimer for the screening of DVT in Asian patients with acute ischemic stroke may be limited. Studies using D-dimer to screen for DVT showed a very low rate of DVT in Asian patients with acute ischemic stroke [20]. Therefore, the use of LEUS may be more appropriate in selected high-risk patients. LEUS for the detection of DVT also has the benefits of no radiation hazard and cost-effectiveness in comparison with computerized tomography venography. High-risk patients are those with a severe neurological deficit or immobilization, and acute ischemic stroke patients with a low D-dimer level should not be presumed to be free of DVT [21]; in our study, more than 60% of patients with DVT showed a normal D-dimer level. Furthermore, performing LEUS on these patients provided additional information on the size, chronicity, and degree of occlusion of the thrombus, which may help in determining the management of DVT in patients with acute ischemic stroke. However, LEUS is observer dependent and still has limitations in detecting DVT in the pelvic or calf area in comparison to computerized tomography venography [12]. Therefore, the evaluation for detecting DVT in acute ischemic stroke patients may be individualized. Furthermore, in those with high risk DVT, prophylaxis with intermittent pneumatic compression, unfractionated heparin or low-molecular weight heparin must be also individualized based on the risk of intracerebral hemorrhage [22].

Our study has several limitations, including those stemming from the small sample size from a single center. The LEUS was performed within 3 days from admission, which is a very early period, and the prevalence of DVT may have increased if the LEUS was performed later on after the index stroke, and the relative power of the predictors could also have been different. Furthermore, we only checked D-dimer on the day after admission, and did not make any follow-up checks. The usefulness of D-dimer at follow-up was observed in a former study [9]. Third, patients with distal DVT, for which the clinical implication is still controversial, were included in this study. Finally, only a limited number of patients received intermittent pneumatic compression, as it was not commonly used during the study period. The results may differ under a more aggressive care to prevent DVT in those with high risk patients.

## Conclusion

Despite these limitations, our study shows that the prevalence of DVT is considerable in Asian patients with acute ischemic stroke. High neurological severity, but not the D-dimer level, was associated with the presence of DVT. Therefore, the role of D-dimer for screening for DVT in Asian patients with acute ischemic stroke may be limited, and LEUS should be considered for those patients with severe neurological deficit or immobilization.

## Data Availability

The datasets used and/or analyzed during the current study are available from the corresponding author on reasonable request.

## Abbreviations

CI: Confidence interval
DVT: Deep vein thrombosis
LEUS: Lower-extremity ultrasonography
mRS: Modified Rankin Scale
NIHSS: National Institutes of Health Stroke Scale
OR: Odds ratio
TOAST: Trials of Org 10,172 in Acute STroke
